# Live Donor Partial Hepatectomy for Liver Transplantation: Is There a Learning Curve?

**Published:** 2010-08-01

**Authors:** R. F. Saidi, N. Elias, D. S. Ko, T. Kawai, J. Markmann, S. Feng, A. B. Cosimi, M. Hertl

**Affiliations:** *Transplantation Unit, Department of Surgery, Massachusetts General Hospital, Harvard Medical School, Boston, MA, USA*

**Keywords:** Liver transplantation;, Live donations, Complications

## Abstract

Background: Donor safety is the first priority in living donor liver transplantation (LDLT).

Objective: To determine the characteristics and outcome of live liver donors who underwent donor hepatectomy from January, 1997 to May, 2007 at Massachusetts General Hospital.

Methods: 30 patients underwent LDLT between January, 1997 and May, 2007 at our institution.

Results: The type of graft was the right lobe (segments 5-8) in 14, left lobe (segments 2-4) in 4, and left lateral sector (segments 2 and 3) in 12 patients. The mean donor age was 36 (range: 26-57) years. The mean follow-up was 48 (range: 18-120) months. No deaths occurred. Overall, 8 (26.6%) patients experienced a total of 14 post-operative complications. Donor complications based on graft type were as follows: left lateral sector (16.7%), left lobe (25%), and right lobe (35.7%). The experience was divided into two periods 1997-2001 (n=15) and 2002-2007 (n=15). Overall complications during 2 periods were 40% and 13.3%, respectively (p<0.001). The incidence of grade III complication also significantly decreased; 66.7% vs 33.3% (p<0.01).

Conclusion: Partial hepatectomy in living donors has a learning curve which appears to be approximately 15 cases. This learning curve is not restricted to the surgeons performing the procedure but involves all aspects of patient care.

## INTRODUCTION

Liver transplantation is the treatment of choice for patients with end-stage liver disease (ESLD). Efforts to increase deceased liver donation have seen only modest successes. Rising rates of death on the waiting list led to the use of more innovative and risky approaches to transplantation, including reduced size and split liver organs and, more recently, living donors [1].

Living donor liver transplantation (LDLT) has emerged successfully to partially relieve the shortage of deceased donor grafts caused by the increasing demands of patients with ESLD. Despite rapid adoption of LDLT by numerous centers, many controversies on donor selection, indications, techniques, and ethics exist [1,2]. Before the widely publicized death in New York City, there were only anecdotal reports about deaths or the need for liver transplantation following the donor procedure [1-4]. The Vancouver meeting then reported 14 deaths worldwide in approximately 7000 LDLT procedures [5]. Although potentially lifesaving for the recipient, LDLT is a unique surgical procedure that subjects a healthy donor to a major surgical procedure without direct therapeutic benefits. We conducted this study to determine the characteristics and outcome of live liver donors who underwent donor hepatectomy from January, 1997 to May, 2007 at Massachusetts General Hospital.

## PATIENTS AND METHODS

All potential donors were evaluated in three phases. Phase 1 comprised a comprehensive history, physical examination and laboratory profile, which includes viral serology and blood type. In phase 2, potential donors were counseled by a nontransplant physician, acting as a donor advocate, who ensured donor commitment, motivation and understanding of the risks involved. A social worker and financial advisor also helped the donor with social or financial issues that may arise during the process of donation. Finally, in phase 3 the donor underwent CT volumetric study and magnetic resonance cholangiography (MRC) or CT cholangiography.

Data were collected on all 30 live liver donors who underwent donor hepatectomy from January, 1997 to May, 2007 at Massachusetts General Hospital.

During this period, our program evolved to improve outcome after live donor hepatectomy. These changes were in all aspects of donor work-up and management. For example, routine mesenteric angiogram was replaced by CT angiography. In one case, donor hepatectomy was aborted as MRC missed a segment IV bile duct which was draining into the right system. This was identified during intra-operative cholangiogram and the donor right hepatectomy was aborted. Since then, we have replaced MRC with CT cholangiography. Other examples are routine application of low central venous pressure during the parenchymal transaction, epidural pain management, early post-operative mobilization and oral diet advancement, aggressive management of phosphorus repletion and respiratory toilet. A percutaneous liver biopsy was not routinely performed before dissection of the hepatic hilum. For operative safety, one or two units of autologous packed red cells were stored before the operation. Operation was planned to guarantee 30%–35% remnant liver volume in the donor. The middle hepatic vein was never taken in right lobe donation.

Standard surgical techniques for LDLT have been previously described [6-9]. All donors underwent intra-operative cholangiography through the cystic duct at the beginning of the procedure. We used Cavitron Ultrasonic Surgical Aspirator (CUSA) and bipolar electrocautery for parenchymal transaction.

For assessing complications, the uniform reporting of adverse outcomes of surgery proposed by Clavien, *et al* [10], was adopted as followed: grade I, deviation from the normal post-operative course but without the need for therapy; grade II, complication requiring pharmacologic treatment; grade III, complication with the need for surgical, endoscopic or radiological intervention (IIIa/b: without/with the need for general anesthesia); grade IV, life-threatening complication requiring intensive care; and grade V, death. Originally developed for general surgical procedures, this system has been widely adopted in liver transplantation for standardization of reporting of complication rates for both donors and recipients.

Values are given as mean and range unless otherwise stated. *Student’s t* test was used to compare means. A p value <0.05 was considered statistically significant.

## RESULTS

Donor characteristics are shown in Table 1. The mean donor age was 36 (range: 26-57) years. Eighty percent of donors were biologically related to the recipients. No mortality occurred. The type of allograft was the right lobe (segments 5-8 without middle hepatic vein) in 14, left lobe (segments 2-4) in four, and left lateral sector (segments 2 and 3) in 12 patients (Table 2).

**Table 1:** Donor characteristics

Mean Age (range)	36 (26-57)
Sex	
Male	24
Female	6
Mean BMI	26
Relatedness to recipient	
Biologically related	
Father	8
Mother	6
Son	5
Brother	3
Uncle	1
Aunt	1
Not biologically related	
Brother-in-law	1
Spouse	2
Friend	3

**Table 2:** Graft characteristics and outcomes

Mean graft weight (range)	
LLS (n=12)	287 (247-350)
LL (n=4)	415 (385-450)
RL (n=14)	838 (621-1100)
Mean EBL (range)	
LLS	391 (300-600)
LL	525 (450-600)
RL	566 (300-1000)
Mean LOS (range)	
LLS	8 (6-12)
LL	7.2 (5-10)
RL	7.7 (5-11)
Complications	
LLS (n=2)	ileus (1), biloma (1)
LL (n=1)	atelectasis (1)
RL (n=5)	atelestasis (n=2), phelebitis (1), GI bleeding (1), biloma (1), wound dehiscence (1), biliary stricture (1), perihepatic fluid collection (1), pleural effusion (1), peripancreatic fluid collection (1), ventral hernia (1)
Number of complication	
0	22
1	5
2	1
3	1
4	1
Readmission	
0	25
1	3
2	1
3	1

LLS: left lateral sector, LL: Left lobe, RL: right lobe, LOS: length of stay

Overall, 8 (26.6%) patients experienced a total of 14 post-operative complications. The mean length of hospitalization (LOS) was 7.5 (range: 5-12) days. Table 2 shows LOS based on the graft type.

Donor complications based on graft type were as follows: left lateral sector (16.7%), left lobe (25%), and right lobe (35.7%). Type and grade of complications are summarized in Tables 2 and 3. The most common complications were respiratory (13%; 3 atelectasis and one pleural effusion which needed drainage) and biliary (10%). All donors with atelectasis responded to aggressive chest physiotherapy. There were three biliary complication; two bilomas which responded to percutaneous drain placement and one biliary stricture which needed two sessions of endoscopic management and stent placement.

**Table 3:** Donor complication and the Clavien’s classification

Grade of Complications: n(%)	Complications (1997-2001) (n)	Complications (2002-2007)
I: 4 (28.6%)	atelectasis (3)	superficial phlebitis (1)
II: 1 (7.1%)	Ileus (1)	0
IIIa: 7 (50%)	Biloma (2), perihepatic fluid collection (1), GI bleeding (1), biliary stricture (1)	peripancreatic fluid collection (1), pleural effusion (1)
IIIb: 2 (14.3%)	wound dehiscence (1)	ventral hernia (1)
IV: 0	0	0
V: 0	0	0

The experience was divided into two periods 1997–2001 (n=15) and 2002–2007 (n=15). Overall complications (Tables 3 and 4) during the two periods were 6/15 (40%) and 2/15 (13%), respectively (p<0.001). The incidence of grade III complication also decreased significantly; 67% *vs* 33% (p<0.01). The total number of complications also dropped from 10 (71%) in the first period to four (29%) in the second period. The number of readmissions also significantly dropped (40% *vs* 13%). Interestingly, the majority of allografts in the first period were left lateral segment (10/15) which changed to right lobe (10/15) in the second period. Despite of this fact, the LOS did not change significantly (8.2 *vs* 7.4 days).

**Table 4:** Outcomes in two different periods

	First 15 cases (1997-2001)	Second 15 cases (2002-2007)
Donors with complications (n=8)	6/15 (40%)	2/15 (13.3%)*
Total complications (n=14)	10 (71.4%)	4 (28.6%)*
Grade III complication (n=9)	6 (66.7%)	3 (33.3%)*
Readmission	6/15 (40%)	2/15 (13.3%)*
LOS	8.2	7.4

LOS: length of stay,

* p<0.05

## DISCUSSION

LDLT has been controversial since its inception. Begun in response to deceased donor organ shortage and waiting list mortality, LDLT was initiated in 1989 in children, grew rapidly after its first general application in adults in the United States in 1998, and has declined since 2002 [1,2]. LDLT still accounts for less than 5% of adult liver transplants, significantly less than in kidney transplantation where living donors account for 40% of all transplantations performed [1]. The ethics, optimal utility, and application of LDLT remain to be defined. In addition, most studies to date have focused on post-transplantation outcomes and have not included the effect of the learning curve on outcome. The initial reports of high recipient successes and low donor morbidity rate led to rapid expansion of adult-to-adult LDLT, and, by 2001, this procedure accounted for more that 400 transplantations (10% of all adult liver transplantations done in the United States that year) [1]. However, following a well-publicized donor death in 2002, rates of adult-to-adult LDLT declined precipitously and have remained in the range of 250–300 per year subsequently [1,2].

The Adult-to-Adult Living Donor Liver Transplantation (A2ALL) cohort study was initiated in 2002 as a cooperative research agreement funded by the National Institute of Health with nine liver transplant centers and a data coordinating center. Across the nine A2ALL centers, the overall donor complication rate was 38% [11]. In A2ALL study, the majority of donors (n=245; 62%) did not suffer any complications, defined by the Clavien classification as any alteration from the ideal post-operative course with complete recovery. However, 148 (38%) donors had a total of 220 complications. Eighty-two (21%) donors had one complication, 40 (10%) had two, 16 (4%) had three, and 10 (3%) had four to seven complications. In our study, eight (27%) donors had a total of 14 complications; five (63%) had one complication, one (13%) had two, one (13%) had three and another one (13%) had four complications.

In A2ALL cohort [11], the most common complication in donors were biliary (9%), bacterial infections (12%), incisional hernia (6%), pleural effusion requiring intervention (5%), neuropraxia (4%), re-exploration (3%), wound infections (3%), and intra-abdominal abscess (2%). Certain individual complications are worth highlighting: Biliary complications have been one of the major concerns in adult LDLT donors and recipients [11-15]. In our study, biliary and respiratory complications were the most common type of donor morbidity, occurring in nearly 13% and 10% of cases, respectively. Incisional hernia only occurred in one (3%) donor. Brachial plexus injury resulting in neuropraxia was also relatively common in A2ALL study and resulted in lasting neuromuscular disability in two donors [11]. This injury usually results from malpositioning on the operating table during a prolonged procedure. In fact, the mean operative time was 455 min for those without neuropraxia and 530 min for those with neuropraxia (p=0.056). This can result in major functional disability, as well as permanent work disability for donors whose occupations depend on motor function of the arm. Fortunately, we did not observe this complication in our donors. Our policy is to always tuck the right arm and have the left arm only 80 degree extended.

Middleton and collegues [16] did a systematic review of literature on the outcome of LDLT. Donor morbidities ranged from 0% to 100%, with a median of 16.1%. Biliary complications and infections were the most commonly reported morbidities. The median reported rate of biliary complications, the most common of which were biliary leaks and biliary strictures, was 6.2%, with reported rates ranging from 0% to 38.6%. Rates of infections, most commonly wound infections, as well as urinary tract infections, pneumonia, and other infections ranged between 0% and 28.6%. The median reported infection rate was 5.8%. 

The Hong Kong group [17,18] reported an overall major complication rate of 14% that was reduced to 6% in their second 50 patients. Minor complications decreased from 26% to 8%. In our study, the overall complication rate, readmissions and grade III complications decreased with experience over time (Tables 3 and 4). We believe that partial hepatectomy in living donors has a learning curve which appears to approximate about 15 cases. This learning curve is not restricted to the surgeons performing the procedure but involves all areas of pre- , peri- and post-operative patient care. Our experience emphasizes that LDLT can be safely provided by active liver transplant programs performing only modest number of LDLT. We have been upholding the policy that donors deserve treatment of the highest standard. We attempted to make the process as safe as possible by constant upgrading the standards of the pre-operative, operative and post-operative care. The standards, as mentioned previously, could be due to many factors attributed to patients, surgeons, anesthesiologists, nurses, and supporting staff.
